# Renal Wounds

**Published:** 1918-09

**Authors:** 


**Renal Wounds.** Chevassu, Le Prog. Med., Feb. 23, observed 46 recent and 10 old wounds. Haemorrhage and infection indicate immediate operation but nephrectomy should not be done unless absolutely necessary. Suture and tamponage usually control the haemorrhage. Old cases rarely show urinary abnormalities.

Bazy, ibid., had removed only one kidney for war wound. He insisted on the superiority of the transverse anterior route of operation. Lapointe believed in intervention in lumbar and lumbo-abdominal wounds but had practiced only two nephrectomies.

